# The Future Is Now: Preparing Sport Management Graduates in Times of Disruption and Change

**DOI:** 10.3389/fspor.2022.813504

**Published:** 2022-03-10

**Authors:** W. James Weese, Michael El-Khoury, Graham Brown, W. Zachary Weese

**Affiliations:** ^1^School of Kinesiology, Western University, London, ON, Canada; ^2^Catapult Career Advantage, University of Windsor, Windsor, ON, Canada

**Keywords:** disruption, higher education, sport management, preparation, COVID-19

## Abstract

COVID-19 disrupted the world, and the impacts have been experienced in many areas, including sport and higher education. Sport management academicians need to reflect on the past two years' experience, determine what worked and what did not work, and avoid the temptation of automatically returning to past practices. The authors of this manuscript applied the disruption literature and propose transformative changes in what sport management academicians teach (e.g., greater emphasis on innovation, entrepreneurship, automation, critical thinking skills to facilitate working in flexible environments and across areas), how colleagues teach (e.g., heightened integration of technology, blended learning models) and where colleagues teach (on-campus and distal delivery modes, asynchronous and synchronous delivery to students on campus and across regions/countries). Examples of start-up companies and entrepreneurial ventures are offered to help illustrate the changing sports landscape and the emerging opportunities for current and future students, graduates, and professors. Sport management professors are offered some suggestions to assist them in seizing this opportunity.

## Introduction

The late Harvard professor Clayton Christensen introduced the concept of “disruptive innovation” to the business literature by describing how nimble and future-oriented organizations did things differently, and in doing so, effectively differentiated themselves from their competitors. These organizations accurately forecasted trends, precisely determined emerging consumer wants and needs, and effectively delivered new or adapted programs and services that heightened their competitive advantage and increased their market share (Christensen and Eyring, 2011; Christensen et al., 2011). Less agile organizations led by leaders who refused to embrace strategic change were negatively impacted and put out of business in some cases. History has provided countless examples of companies and industries that have followed this course. Estrin (2015) chronicled one of the most poignant examples of a company not paying attention to the changing times in the example of Kodak. This company was once the industry leader in the field of photography. According to Estrin (2015), Steve Sasson, a young engineer, pitched the futuristic idea of the digital camera to the firm in 1975. Leaders summarily dismissed the idea and quickly pointed to Kodak's leadership position in the film and image reproduction areas. Unfortunately, they missed the bigger picture, and when Fuji and Nikon developed their digital camera 10 years later, Kodak paid the price. Five years after this launch, Kodak was out of business (Estrin, 2015).

Other examples of industries being disrupted can be observed in how Nike trumpeted Reebok in the athletic apparel and footwear fields or how Netflix transformed the video rental business with electronic delivery that quickly put Blockbuster out of business. Think of the impact that both Uber and Airbnb have had on the ride-sharing and hotel industries. Organizations must anticipate changes in their industry and adapt their strategies and practices. Failure to do so puts them at risk of being left behind. COVID-19 has accelerated the need for industries and their practices to adapt (Huber and Sneader, 2021).

Industry leaders must also embrace technological advancements. Despite their recent entries into the marketplace, organizations that have embraced innovation and technology have redefined their industries and are thriving (e.g., Amazon, Shopify, Google, Uber, Airbnb, Skip the Dishes). Huber and Sneader (2021) suggested that some of the practices forced on providers and consumers during COVID-19 will remain long after the pandemic subsides. They offered examples like telehealth, e-commerce, and heightened use of automation as examples of the changes necessitated by the pandemic, but likely to become standard practice.

Furthermore, the start-up/venture capitalist culture has risen in the 21st century and has disrupted many markets (Christensen, 2003). A start-up company is defined as a newly founded organization or entrepreneurial venture in the beginning phases of development (Cannone and Ughetto, 2014). These organizations are nimble, meet a need, and are adaptable (Robehmed, 2013). According to Lee (2016), they have a different organizational culture than traditional organizations. They require less “bricks and mortar” infrastructure and rely more on spaces that facilitate idea generation, heightened synergy, and technology interfaces for remote collaborations (Lee, 2016). These characteristics appeal to many recent graduates seeking an appropriate blend of challenge and freedom in their work experiences (Gabrielson, 2019). Given the disruptive forces impacting sport, they may prove to be a growth area for sport management graduates.

## Disruptive Impacts on Sport

Significant changes are taking place in how society engages in sport as participants and spectators. Attendance at some professional sporting events has been in decline in many markets over the past decade (Stebbins, 2017; Damgaard, 2018; Suneson, 2019), and it typically comprised of older fans (Bryne, 2020). COVID-19 significantly altered attendance patterns, and as some suggest, permanently (Ratten, 2020; Wilson, 2021). Many sports leagues were shut down, and others were required to operate with limited numbers of spectators. Out of necessity, fans were forced to consume sport through television and social media vehicles (Goldman and Hedlund, 2020; Hull and Romney, 2020). Mastromartino et al. (2020) suggested that broadcasting and social media advancements have enriched and transformed the fan experience. Will fans return to their previous ways of physically attending games once the pandemic subsides? Some (Mastromartino et al., 2020; Ratten, 2020; Wilson, 2021) suggest that many will not.

Given the consumption pattern shifts and the economic consequences of COVID-19, it is reasonable to assume that the traditional size of the sports organizations that previously employed our graduates will be smaller, and those working in these organizations might be required to do more with less. Some graduates may need to assume new or expanded roles. Current and future graduates will need to be critical thinkers, flexible, adaptable, and confident working across disciplinary areas. Some may wish to strike out on their own and use their entrepreneurial backgrounds to create their own employment (Escamilla-Fajardo et al., 2020). Some may find employment in alternative settings like start-up companies. These realities point to the undeniable fact that sport management students will need a new kind of education—one that prepares them to be highly adaptable, innovative, and progressive. They will need to be entrepreneurial. They will need to understand automation (Johnson, 2020) and the impacts that technological advancements have on our field, and their employment prospects.

## The Sport and Technology Connection

Technology and sport have become increasingly dependent on each other during the COVID-19 period. The authors of this manuscript and others (e.g., Readwrite., 2018; Pizzo et al., 2018; Proman, 2019; Reitman et al., 2019; Finch et al., 2020) predict that technology will exponentially increase in the coming years create boundless opportunities for progressive leaders in sport management. This scenario may be especially true for those who embrace start-up industries in sport (e.g., esport), which will use technology to keep fixed costs low and penetrate emerging markets (Finch et al., 2020).

The start-up company concept originated in the Silicon Valley in the 1980s (Larsen and Rogers, 1984). According to Fontinelle (2020), start-up companies emerged to develop and deliver unique products or services that could more effectively meet the needs of the marketplace. These companies typically started small before expanding into larger enterprises. Some of today's leading companies (e.g., Amazon, Shopify, Microsoft, McDonald's, Apple) began as start-up companies.

However, according to Au (2017), the sports marketplace is one of the more difficult sectors for new brands to integrate. In Canada, there are only a few incubators and sports laboratories to support start-up companies. Some, like Ryerson University's *Future of Sport Lab*, is a joint effort between the university and Maple Leaf Sport and Entertainment (MLSE) and is an incubation hub that supports research and innovation that often leads to partnerships with private or public funding groups (Start-up Here Toronto, 2019). The University of Guelph proudly supports the International Institute for Sport Business and Leadership (n.d), a start-up that brings academic and industry leaders together to identify and pursue action research projects. These kinds of programs represent the new thinking that is required in sport management. The emergence of other start-up companies in sport highlights the explosive growth of this area.

Virtual Reality (VR) and, in particular, esports represent a rapidly growing segment in the sports industry (Jonasson and Thiborg, 2010; Funk et al., 2018; Collis, 2020), and by extension, an area that sport management scholars should integrate into their teaching and research programs. While initially designed for children and youth, interest and participation have also spawned into older populations. According to Clement (2021), there were 2B world-wide video gamers in 2015, and the number is expected to grow to 3B by 2023. The Canadian Sport Daily (2020) supported this growth prediction by reporting that there were 2.7B worldwide video gamers by the end of 2020. Alton (2019) noted similar growth in viewership of competitive gaming events. She noted that esports had a world-wide fan base in excess of 454M, up from 380M in 2018 (Willingham, 2018) and was experiencing a growth rate of a 14% per year. In comparison, and prior to the onset of pre-COVID-19, the NCAA Men's “March Madness” Basketball Tournament had viewership that maximized at 28M (Wilson, 2021). Imagine the advertising and branding opportunities esports provides corporations looking to reach a young and emerging market. Some speculate that esports games will soon be included in major international events, such as the Asian Games in 2022 and the Paris Olympics in 2024 (Kocadag, 2019). The future for esport is bright (Mulcahy, 2019). Advancements in, and access to technology will fuel future growth. The same could be said for another growth area in sport, namely, legalized gambling. Online sports gambling is proving to be a highly profitable and permanent fixture impacting sports spectatorship. Are sport management scholars also discussing these developments in their classrooms, and are they preparing graduates to compete in these types of emerging areas? Sport and sport management have been disrupted, and as noted below, so have the institutions traditionally preparing sport management graduates and leaders of the future.

## Disruptive Impacts on Higher Education

Kak (2018) and Levin (2021) have called for significant change in higher education for some time. They argued that the 20th-century models need to be updated in terms of what is taught and how it is delivered. Automation, artificial intelligence, hologram technology, and advances in telecommunications offer unlimited opportunities for changing how academic programs can be constructed and delivered.

Govindarajan et al. (2021) suggested that COVID-19 has accelerated the change process. As a result of COVID-19, lecture theaters and campuses were abandoned, and professors were forced to integrate technology and implement remote teaching strategies for their students. Naturally, there were bumps along the way given this sudden shift. Professors and students both claimed to have missed the relationship-building aspects that in-person delivery offers to support and inspire learning. However, while many students and professors struggled with this adaptation and longed for pre-pandemic practices, some students and professors thrived in this new environment. Many would like some of these new practices to continue. Some students have reported that they liked the pace and flexibility of taking their classes remotely and in an asynchronous format. Many stayed at home and saved money previously spent on transportation, accommodations, and parking. Professors found that the heightened use of technology could enrich their courses. Small group discussions could be effectively facilitated through virtual chat rooms. Professors could integrate internationally-renowned experts into their courses who didn't need to travel to deliver guest lectures. In some sectors of our campuses, productivity increased. The pandemic proved that there were other ways of delivering higher education, and once again, necessity proved to be the “mother of invention.”

As a result, Levin (2021) and Govindarajan et al. (2021) encouraged professors and program leaders to reflect deeply on the needs of students, the content of courses, and be open to adopting some of the practices that had to be implemented during the pandemic. Perhaps programs, courses, or parts of each could be more effectively delivered in virtual or a hybrid of virtual and face-to-face formats. They made a case for progressively integrating more digital technologies (e.g., remote delivery, holograms) to enrich learning. Some students may prefer the benefits of remote delivery (or perhaps some combination of time on campus and time in remote delivery modes). If this delivery option exists, new cohorts of students might be drawn to the sector. Recognizing the benefits and cost savings of some remote learning, in whole or part, might prompt some institutions to reduce their infrastructure footprint. Some campuses could adopt a blended model where students in the first and final years have an on-campus experience, while those in the middle years consume their programs from a remote setting. Some of the more reputable institutions may take this opportunity to significantly expand their high-demand programs previously restricted by space realities.

Think of the cost savings for some students if they did not have to be on campus for their entire university experience. Think of program expansion opportunities if courses, programs, or parts of programs could be delivered through distance education. Consider the cost savings if universities could more discriminately rationalize program offering and efficiently share courses or parts of programs with other institutions. Incremental revenue could be generated from selling or renting some freed-up land or buildings. The high costs of constructing and operating facilities could be reduced. Program officials could offer more courses in asynchronous formats so students could consume their courses at a pace and time that is advantageous to them. Academic leaders and governing boards more effectively future-proof higher education by adopting some of these practices. Fiscal realities and societal pressure might demand such action.

It is a challenging time, and neither sport nor sport management educational programs are immune from the disruptive forces and seismic changes outlined above. Graduates are now entering employment opportunities that are less structured, more fluid, and less permanent (Vedder et al., 2013). The situation has been exponentially accelerated by the economic and labor market disruptions of COVID-19 (Gentilini et al., 2020). Bold questions must be addressed. Are colleagues delivering what sport management students need? Are they preparing graduates to be thought leaders who are entrepreneurial, independent, and confident to navigate careers in times of rapid societal change? Are graduates critical thinkers who can adapt and work across a number of areas given the anticipated smaller workforces? Is the content of sport management programs cutting-edge and progressive? Are the tuition and related educational cost structures for students realistic and affordable given the market forces, value propositions, and economic times (i.e., current and predicted)? Sport management colleagues must adapt to thrive given the changes taking place in sport, sport management, and higher education (Christensen and Eyring, 2011; Christensen et al., 2011).

## Conclusion

COVID-19 has been a devastating virus that has disrupted society in innumerable ways. It will also have far-reaching implications on institutions of higher learning and in the ways that society participates or consumes sport. Organizations that prevail will be nimble, progressive, and innovative. Implementing some or all of the suggestions outlined below could improve and modernize our academic programs. Colleagues can lead change by:

1. Developing and delivering a curriculum that covers the traditional areas like leadership, finance, economics, analytics, as well as the emerging areas in the field like innovation, entrepreneurship, automation, artificial intelligence, and start-up companies.
2. Expanding experiential learning opportunities for students beyond the traditional sport settings and include opportunities in emerging organizations like start-up companies. Students need to understand the rules of engagement in emerging technologies and the realities of working in agile, risk-taking ventures.
3. Implementing higher levels of technology into the curriculum to bring world experts into the digital classrooms. Industry leaders (from across the globe) can be beamed into digital classrooms with minimal expense via Zoom or hologram technologies. Technological advancements allow for virtual meeting rooms where smaller groups of students can have deeper discussions and reflection sessions.
4. Using technology to share courses and professors between campuses and expanding digital platforms to reach more students in synchronous and asynchronous formats. Many universities are facing fiscal challenges. Courses between campuses could be shared to enrich the experience and preparation of students at little or no cost to the host institutions. Sport management could be leaders in this synergistic approach.
5. Ensuring that guest speakers, case studies, and classroom examples are drawn for diverse fields (e.g., start-up companies, venture capitalists, e-sports, gaming, fantasy sports, sports gambling) in addition to those from traditional sports settings.
6. Expanding experiential learning opportunities for students by investing in case competitions that include examples from start-up companies and other emerging areas in the field. These rich learning opportunities allow students the opportunity to apply course content and, if also drawn from emerging industries, can help keep the program current. To increase application and underscore relevance, have practitioners pose the challenge question and involve them in evaluating the proposed solutions.

The impact of COVID-19 has accelerated the need for change in sport, sport management and in the institutions that house sport management educational programs. Sport management colleagues are encouraged to reflect on the suggestions outlined above and ensure that the programs delivered to students align with the current and emerging developments in the industry and in higher education.

## Data Availability Statement

The original contributions presented in the study are included in the article/supplementary material, further inquiries can be directed to the corresponding author.

## Author Contributions

JW took the lead in the preparation of this article. ME-K, GB, and ZW are former students and sport management leaders who have experience in the industry, recognize the disruption that has taken place in recent years, and have experience working with recent graduates of our sport management programs, and provided helpful insights and examples that have been integrated into this article. All authors contributed to the article and approved the submitted version.

## Conflict of Interest

GB was employed by company Catapult Career Advantage. The remaining authors declare that the research was conducted in the absence of any commercial or financial relationships that could be construed as a potential conflict of interest.

## Publisher's Note

All claims expressed in this article are solely those of the authors and do not necessarily represent those of their affiliated organizations, or those of the publisher, the editors and the reviewers. Any product that may be evaluated in this article, or claim that may be made by its manufacturer, is not guaranteed or endorsed by the publisher.
